# SPECT/CT with radiolabeled somatostatin analogues in the evaluation of systemic granulomatous infections

**DOI:** 10.1590/0100-3984.2016.0076

**Published:** 2017

**Authors:** Paulo Henrique Silva Monteiro, Thiago Ferreira de Souza, Maria Luiza Moretti, Mariangela Ribeiro Resende, Jair Mengatti, Mariana da Cunha Lopes de Lima, Allan Oliveira Santos, Celso Darío Ramos

**Affiliations:** 1 Division of Nuclear Medicine, Department of Radiology, School of Medical Sciences, State University of Campinas (Unicamp), Campinas, SP, Brazil.; 2 Division of Infectology, Department of Internal Medicine, School of Medical Sciences, State University of Campinas (Unicamp), Campinas, SP, Brazil.; 3 Nuclear Energy and Research Institute (IPEN), São Paulo, SP, Brazil.

**Keywords:** Single photon emission computed tomography, X-ray computed tomography, Octreotide, Gallium-67, Tuberculosis, Paracoccidioidomycosis, Tomografia computadorizada por emissão de fóton único, Tomografia computadorizada por raios X, Octreotide, Gálio-67, Tuberculose, Paracoccidioidomicose

## Abstract

**Objective:**

To evaluate SPECT/CT with radiolabeled somatostatin analogues (RSAs) in systemic granulomatous infections in comparison with gallium-67 (^67^Ga) citrate scintigraphy.

**Materials and Methods:**

We studied 28 patients with active systemic granulomatous infections, including tuberculosis, paracoccidioidomycosis, pneumocystosis, cryptococcosis, aspergillosis, leishmaniasis, infectious vasculitis, and an unspecified opportunistic infection. Of the 28 patients, 23 had started specific treatment before the study outset. All patients underwent whole-body SPECT/CT imaging: 7 after injection of ^99m^Tc-EDDA-HYNIC-TOC, and 21 after injection of ^111^In-DTPA-octreotide. All patients also underwent ^67^Ga citrate imaging, except for one patient who died before the ^67^Ga was available.

**Results:**

In 20 of the 27 patients who underwent imaging with both tracers, 27 sites of active disease were detected by ^67^Ga citrate imaging and by SPECT/CT with an RSA. Both tracers had negative results in the other 7 patients. RSA uptake was visually lower than ^67^Ga uptake in 11 of the 20 patients with positive images and similar to ^67^Ga uptake in the other 9 patients. The only patient who did not undergo ^67^Ga scintigraphy underwent ^99m^Tc-EDDA-HYNIC-TOC SPECT/CT-guided biopsy of a lung cavity with focal RSA uptake, which turned to be positive for aspergillosis.

**Conclusion:**

SPECT/CT with ^99m^Tc-EDDA-HYNIC-TOC or ^111^In-DTPA-octreotide seems to be a good alternative to ^67^Ga citrate imaging for the evaluation of patients with systemic granulomatous disease.

## Abstract (pt)

**Objetivo:**

Avaliar o estudo SPECT/CT com análogos de somatostatina radiomarcados (RSA) em infecções granulomatosas sistêmicas, em comparação com o estudo com gálio-67 (^67^Ga).

**Materiais e Métodos:**

Vinte e oito paciente com infecção granulomatosa sistêmica ativa foram estudados, incluindo tuberculose, paracoccidioidomicose, pneumocistose, criptococose, aspergilose, leishmaniose, vasculite infecciosa e uma infecção oportunista inespecífica. Vinte e três tinham iniciado o tratamento previamente ao estudo. Todos fizeram imagem de varredura e SPECT/CT, 7 deles pós-injeção de ^99m^Tc-EDDA-HYNIC-TOC e os outros 21 pós-injeção de ^111^In-DTPA-octreotide. Todos os pacientes também fizeram cintilografia com ^67^Ga, exceto um, que fez biópsia guiada por ^99m^Tc-EDDA-HYNIC-TOC.

**Resultados:**

Vinte e sete sítios de atividade foram detectados com ^67^Ga em 20 de 27 pacientes, também vistos nos estudos com RSA. Ambos foram negativos nos outros 7 pacientes. A captação de RSA foi visualmente menor que a de ^67^Ga em 11 de 20 pacientes positivos e similar nos outros 9. Um paciente que não pôde fazer cintilografia com ^67^Ga, fez biópsia guiada por ^99m^Tc-EDDAHYNIC-TOC SPECT/CT em uma cavidade pulmonar com captação desse traçador, que foi positiva para aspergilose.

**Conclusão:**

SPECT/CT com ^99m^Tc-EDDA-HYNIC-TOC ou ^111^In-DTPA-octreotide parece ser uma boa alternativa para o estudo com ^67^Ga na avaliação de pacientes com doença granulomatosa sistêmica.

## INTRODUCTION

Octreotide is a somatostatin analogue with several clinical applications, such as controlling upper gastrointestinal bleeding and treating carcinoid syndrome^(1)^. When radiolabeled, it becomes an important diagnostic and therapeutic tool for neuroendocrine tumors. It was initially labeled with indium-111 (^111^In), as ^111^In-DTPA-octreotide^(2)^. Subsequently, tyrosine-octreotide radiolabeled with metastable technetium-99 (^99m^Tc-EDDA-HYNIC-TOC) became available and was shown to have several advantages over ^111^In-DTPA-octreotide, including lower cost, ease of storage in a freeze-dried kit for labeling with ^99m^Tc, better image quality, and a faster image acquisition protocol^(3,4)^.

When activated, monocytes and lymphocytes show strong membrane expression of somatostatin receptors^(5,6)^. Therefore, we expected to detect marked expression of those receptors, measurable by radiolabeled somatostatin analogue (RSA) scintigraphy, in granulomas and activated leukocytes that manifested as an immune response to systemic infectious diseases, such as tuberculosis, paracoccidioidomycosis, histoplasmosis, opportunist infections, pneumocystosis, and aspergillosis^(7)^. Those diseases, as a group, have a high prevalence in many developing countries and a high number of new cases per year. Owing to the nature of their physiology, these diseases tend to spread throughout the whole body. The treatment is usually slow and often empirical, especially because there are only a few reliable methods to evaluate the disease activity before, during, and after treatment^(8-10)^.

Conventional computed tomography (CT) can produce false-negative results for disease activity during and after treatment^(11,12)^. Gallium-67 (^67^Ga) scintigraphy has long been used in order to detect and evaluate systemic granulomatous infections and is in fact considered as the gold standard for evaluating such infections^(13,14)^. However, it is difficult to use ^67^Ga in many situations, because it must be ordered days ahead of the moment of the procedure and, in many countries, only on specific days of the week. In addition, in specific situations, it is necessary to acquire images at 48-72 h after injection, which limits its use in urgent and acute care.

The objectives of this study were to evaluate RSA uptake in patients with systemic granulomatous infections and to compare it with ^67^Ga citrate uptake, which was used as the reference method.

## MATERIALS AND METHODS

This was an observational, prospective study. The following inclusion criteria were applied: having a confirmed diagnosis, either clinically or according to laboratory test results, of a systemic infectious disease (such as tuberculosis, paracoccidioidomycosis, histoplasmosis, opportunistic infections, pneumocystosis, and aspergillosis); having been referred to our facility for ^67^Ga citrate scintigraphy; being under regular inpatient or outpatient follow-up of infectious diseases; and being 18 years of age or older. Patients who were pregnant or breastfeeding were excluded, as were those who declined to submit to or did not tolerate either of the imaging procedures at any time.

All procedures were performed in accordance with the ethical standards of the institutional and national research committees and with the 1964 Helsinki declaration and its later amendments or comparable ethical standards. All participating patients gave written informed consent.

We studied 28 consecutive patients (11 females), with a mean age of 43.2 ± 14.9 years (range, 18-76 years), all of whom had active systemic granulomatous infection: tuberculosis (in 13); paracoccidioidomycosis (in 8); pneumocystosis (in 2); cryptococcosis (in 1); angioinvasive pulmonary aspergillosis (in 1); leishmaniasis (in 1); *Bartonella henselae* infection-associated vasculitis (in 1); and an unspecified opportunistic infection, treated empirically as tuberculosis (in 1). Of the 28 patients, 23 had already started specific treatment for the diverse infectious diseases an average of 19 ± 23 days before their inclusion in the study. The aspergillosis, cryptococcosis, and pneumocystosis patients were immunocompromised. Almost all of the patients had lung disease, except for the patient with *B. henselae* infection-associated vasculitis, who had diffuse large-vessel disease.

All patients were assessed through clinical interviews and examinations, as well as through imaging studies with ^99m^Tc-EDDA-HYNIC-TOC or ^111^In-DTPA-octreotide, depending on radiopharmaceutical availability. All patients were also submitted to ^67^Ga citrate imaging, except for one patient who died before the radiotracer became available. In that patient, the ^99m^Tc-EDDA-HYNIC-TOC imaging findings were correlated with those of a guided biopsy. The interval between imaging sessions did not exceed 10 days (range, 4-10 days). For all patients, current diseases, known disease activity foci, disease duration, disease progress, disease severity, and responses to previous therapies were evaluated in the clinical interviews and by reviewing patient charts.

Scintigraphy with ^99m^Tc-EDDA-HYNIC-TOC was performed in a SPECT/CT system (Symbia T2; Siemens, Erlangen, Germany) with a low-energy, high-resolution collimator. The radiotracer dose was 249-370 MBq, corrected for patient body weight, with a mean dose of 300 MBq. In all patients, whole-body SPECT/CT scanning (table speed, 6 cm/min) was performed at 4 h after injection and was followed by additional SPECT/CT scans (matrix, 64 × 64 pixels; 360° detector configuration, with a rotation of 180° for each detector; 6° per step; 30 s per view; and a 2-slice CT with voltage and current set at 130 kV and 35 mA, respectively) of regions of interest selected by an analysis of the whole-body scan. In some patients, additional planar images (1,000,000 counts per static image) were acquired 24 h after radiotracer injection, when it was deemed necessary in order to clarify inconclusive imaging findings or to resolve discrepancies between the clinical data and the images.

Scintigraphy with ^111^In-DTPA-octreotide was performed in the same SPECT/CT system described above, with a high-energy collimator and a radiotracer dose of 185 MBq. In all patients, whole-body SPECT/CT scanning (table speed, 3 cm/min) was performed 24 h after injection and was followed by additional SPECT/CT scans (matrix, 64 × 64 pixels; 360° detector configuration, with a rotation of 180° for each detector; 6° per step; 35 s per view; and a 2-slice CT with voltage and current set at 130 kV and 35 mA, respectively) of regions of interest selected by an analysis of the whole-body scan.

As previously stated, all but one of the patients underwent ^67^Ga scintigraphy. The ^67^Ga scintigraphy was performed in the same SPECT/CT system described above, with a high-energy collimator and a radiotracer dose of 185 MBq. In all patients, whole-body SPECT/CT scanning (table speed, 8 cm/min) was performed 48 h after injection and was followed by additional SPECT/CT scans (matrix, 64 × 64 pixels; 360° detector configuration, with a rotation of 180° for each detector; 6° per step; 30 s per view; and a 2-slice CT with voltage and current set at 130 kV and 35 mA, respectively) of regions of interest, selected by an analysis of the whole-body scan.

## RESULTS

**Figure 1:**
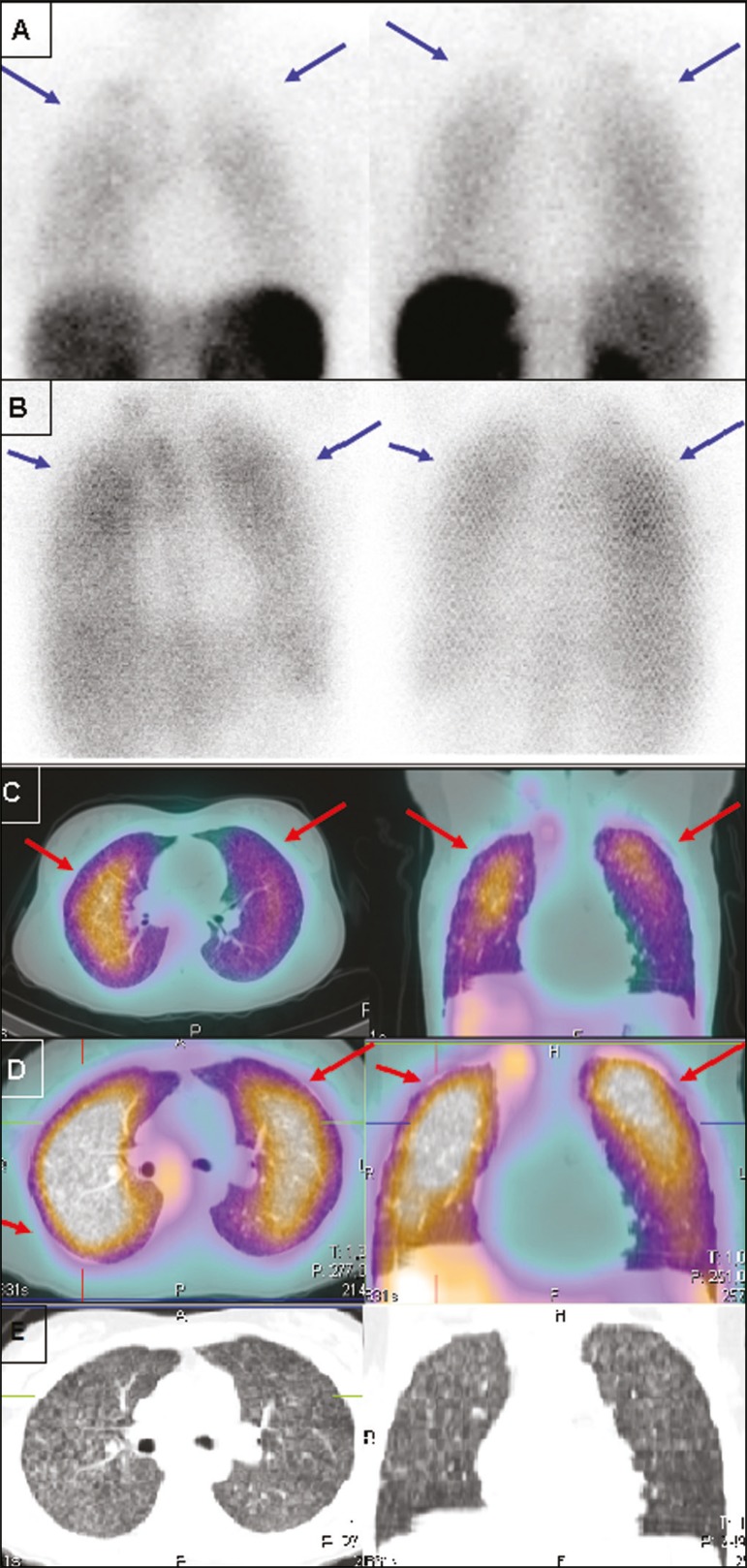
Static lung images (A,B) and transaxial SPECT/CT images (C,D) with ^99m^Tc-EDDA-HYNIC-TOC (A,C) and ^67^Ga citrate (B,D) of an 18-year-old patient with miliary tuberculosis, plus the CT imaging (E) performed for the scan (2-slice CT, obtained during respiration to avoid mismatching artifacts with the SPECT image). Note the diffuse lung uptake, a pattern typical of miliary tuberculosis, detected on both exams (blue arrows on static images, red arrows on SPECT/CT imaging).

**Figure 2:**
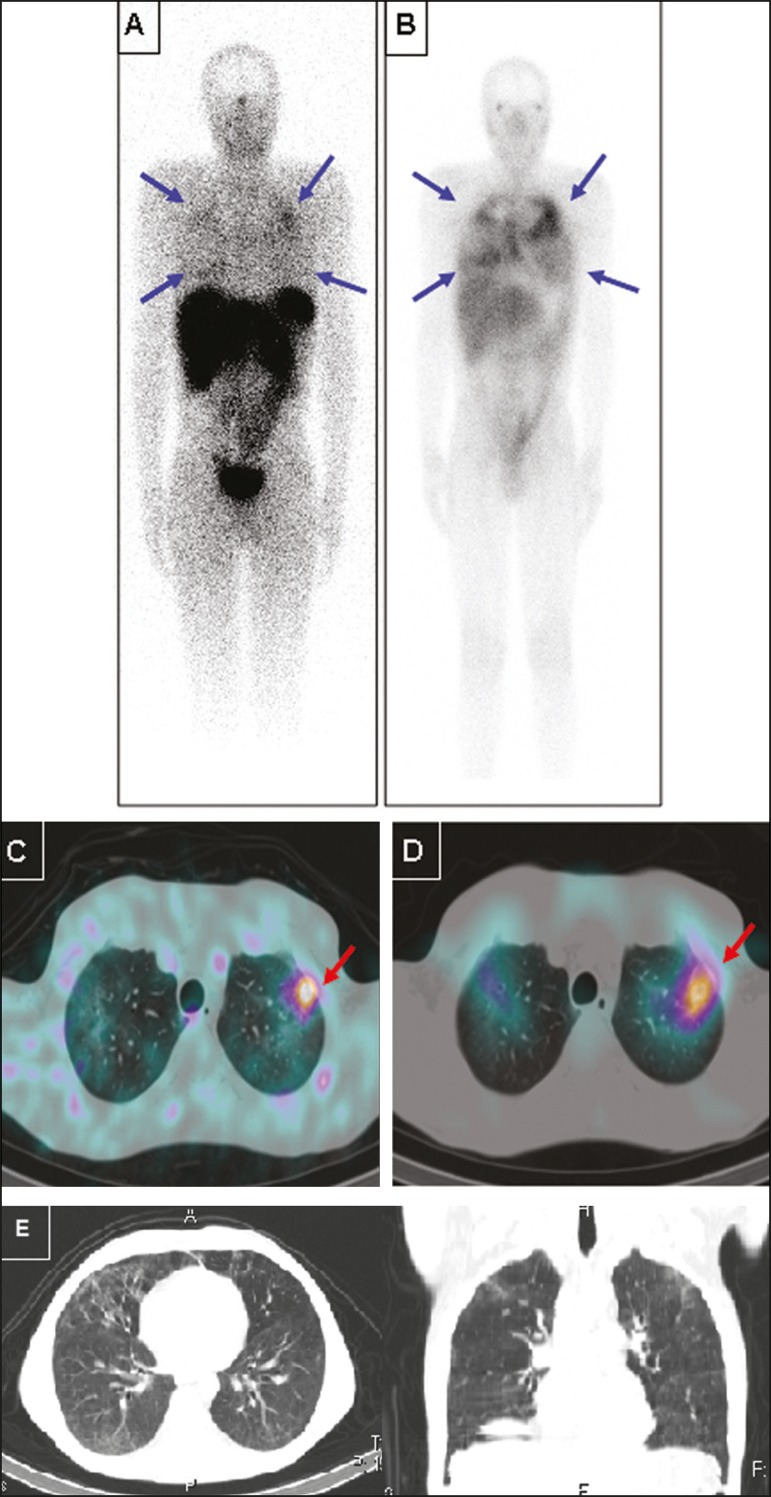
Anterior whole-body images (A,B) and transaxial SPECT/CT images (C,D) with ^99m^Tc-EDDA-HYNIC-TOC (A,C) and ^67^Ga citrate (B,D) of a 39-year-old patient with pneumocystosis. Note the heterogeneous increased uptake in both lungs, more intense on ^67^Ga citrate images (blue arrows). A focal area of more active disease in the left upper lobe is evident with both tracers, and is more clearly seen with SPECT/CT images (red arrows). Selected axial and coronal CT images are also displayed on (E), showing multiple areas of groundglass opacities.

**Figure 3:**
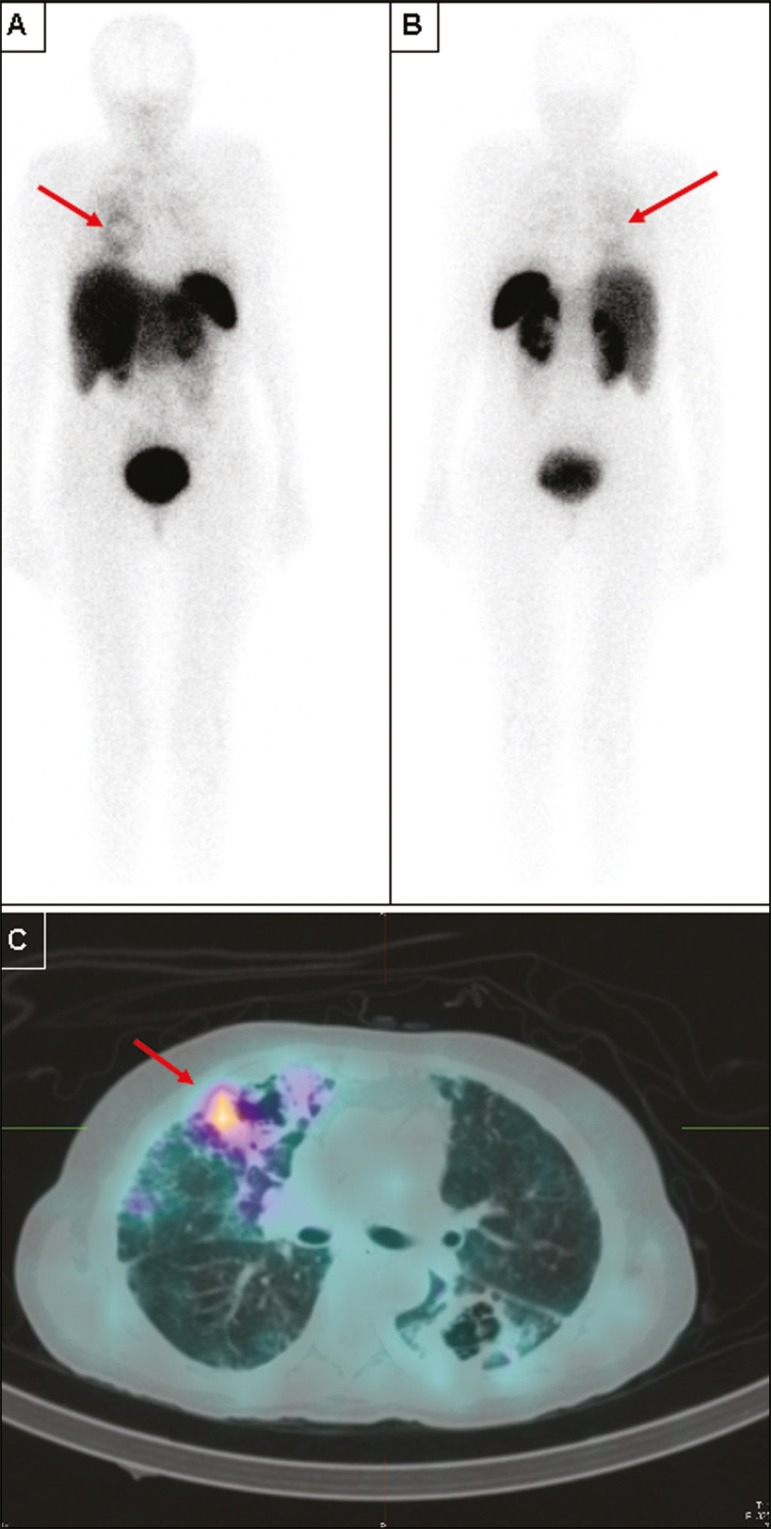
Anterior (A) and posterior (B) whole-body imaging and transaxial SPECT/CT image (C) with ^99m^Tc-EDDA-HYNIC-TOC of a 51-year-old patient with stage C3 HIV infection (AIDS) and an undiagnosed opportunistic respiratory infection. After all serology studies, cultures, and usual examinations were negative for an etiology, it was decided to submit the patient to an unguided lung biopsy. On the day before the biopsy, she was brought to our attention, and ^67^Ga was unavailable in our service, prompting us to suggest ^99m^Tc-EDDA-HYNIC-TOC imaging to guide the biopsy. Whole-body imaging, correlated with SPECT/CT, pointed to a single foci of more significant activity (red arrows), among several other inactive or barely active cavities, as seen on the SPECT/CT imaging. The biopsy was then guided to this cavity, and revealed *Aspergillus sp.*, indicating angioinvasive pulmonary aspergillosis as the cause of the infection. However, due to the severity of the disease, the patient died before it was possible to perform ^67^Ga imaging.

Twenty-seven sites of focal or diffuse active infectious disease were detected by ^67^Ga in 20 of the 27 patients submitted to ^67^Ga scintigraphy. All of those sites were also detected in RSA images (Figure 1). The images obtained with both tracers were negative in the other 7 patients. The intensity of ^67^Ga uptake was visually mild in 5 patients, moderate in 11, and marked in 4. The intensities of ^99m^Tc-EDDA-HYNIC-TOC and ^111^In-DTPA-octreotide uptake in the lesions were visually mild in 13 patients, moderate in 5, and marked in 2. The intensity of RSA uptake was visually lower than ^67^Ga uptake in 11 of the 20 patients with positive images (Figure 2), which was best visualized in SPECT/CT. In the 9 remaining patients, the RSA uptake in the sites of active disease was similar to ^67^Ga uptake. The only patient who did not undergo ^67^Ga scintigraphy was submitted to ^99m^Tc-EDDA-HYNIC-TOC SPECT/CT-guided biopsy of a right-lung cavity with moderate focal uptake. That patient had multiple lung cavities and was under suspicion of having an undiagnosed opportunistic infection, dying before ^67^Ga was available for injection. The lung biopsy revealed aspergillosis (Figure 3).

## DISCUSSION

For over 40 years, ^67^Ga citrate has been successfully used for the diagnosis and follow-up of diverse granulomatous diseases. However, its high cost, limited availability, and complicated imaging logistics limit its use at many nuclear medicine centers, especially centers in developing countries, which are precisely those at which the prevalence of infectious granulomatous diseases is highest. Therefore, there is a need for an alternative to ^67^Ga for the study of these diseases. In a pilot study, our group determined the potential of using RSA in that context^(15)^. The results of the present study demonstrate concordance between RSA and ^67^Ga uptake sites in systemic granulomatous infections, suggesting that RSA scintigraphy could be used as an alternative method to detect disease activity and guide biopsies in the setting of clinically suspected granulomatous disease.

Of particular interest is the case of the patient who did not manage to undergo ^67^Ga scintigraphy (Figure 3). That 51-year-old female patient presented a stage C3 HIV infection (AIDS) and several previous episodes of tuberculosis, which left her with multiple lung cavities. She was brought to our attention while suffering from a new, severe opportunistic infection, which did not respond to various empirical treatments, and her laboratory test results had not revealed the etiology of the infection. She was scheduled to undergo an unguided lung biopsy as a last-ditch effort to find the cause of the disease. She was included as part of our protocol, and her RSA imaging revealed moderate focal uptake in one specific cavity, in the right lung. The SPECT/CT image was then used to guide the lung biopsy, which identified the etiology as *Aspergillus sp*. Although the patient, due to disease progression, did not survive long enough for ^67^Ga to become available, we believe this case illustrates our intended purpose for this method: to quickly locate and direct the investigation of infectious granulomatous diseases. The method can detect which lesions identified by CT represent active infection, as opposed to residual scar tissue. That seems to be especially useful in patients with multiple prior treatments and suspected active infection, as well as in guiding further biopsies to the most active lesions.

We also found limitations to the method investigated in the present study. The intensity of RSA uptake is less marked than is that of ^67^Ga and might require nuclear medicine practitioners who are more experienced in order to read the images. In addition, in our limited sample, RSA imaging did not reveal any additional lesions when compared with ^67^Ga, suggesting that the former is not superior in terms of sensitivity. On the contrary, RSA sensitivity is potentially inferior to that of ^67^Ga, because the lesions tended to uptake less RSA than ^67^Ga. Furthermore, the aforementioned previous treatment might have lowered the sensitivity of both imaging techniques, as might have the 4-10 day interval between the two procedures. However, because the treatment for granulomatous diseases usually takes weeks or months, we do not believe that this was a critical factor. The most severe limitation of our study is the small size of our sample, especially the limited number of patients with each individual disease. Further studies, with larger samples, are needed in order to gather stronger evidence to support the use of RSA scintigraphy for diagnosing these diseases in clinical practice. That is a worthwhile pursuit, given the wider availability, reduced cost, and faster imaging of RSA scintigraphy when compared with ^67^Ga citrate scintigraphy, which is still the current gold standard for the functional imaging of these diseases in many countries.

## CONCLUSIONS

SPECT/CT with ^99m^Tc-EDDA-HYNIC-TOC or ^111^In-DTPA-octreotide seems to be a good alternative to ^67^Ga citrate scintigraphy for the evaluation of patients with systemic granulomatous infections, especially for detecting which lesions found on CT represent active infection, as opposed to residual scar tissue. That seems to be especially useful in patients with multiple prior treatments and suspected active infection, as well as in guiding biopsies to the most active lesions. However, further studies are needed in order to gather more evidence to support its use in diagnosing such diseases in clinical practice.
